# Feasibility study of U-Net-based automatic segmentation of pelvic bone marrow for postoperative radiotherapy in cervical cancer

**DOI:** 10.3389/fonc.2025.1612984

**Published:** 2026-01-27

**Authors:** Hao Qiu, Qianjin Shi, Tianhong Tang, Kang Shen, Yan Zhuang

**Affiliations:** Department of Radiation Oncology, Siyang Hospital, Suqian, China

**Keywords:** U-Net, cervical cancer, organs at risks, automatic segmentation, bone marrow depression

## Abstract

**Objective:**

To investigate the feasibility and clinical value of RT-Mind, a convolutional neural network (CNN)-based auto-segmentation software, in delineating clinical target volume (CTV) and pelvic bone marrow (PBM) as organs at risk (OARs) during postoperative radiotherapy for cervical cancer.

**Methods:**

A retrospective analysis was conducted on 55 cervical cancer patients who underwent postoperative radiotherapy between March 2024 and January 2025. Manual delineations by experienced radiation oncologists were compared with auto-segmentations generated by RT-Mind for CTV and OARs (including rectum, bladder, bowel bag, femoral heads, and bone marrow). Evaluation metrics included Dice Similarity Coefficient (DSC), Hausdorff Distance (HD), Jaccard Index (JAC), and Sensitivity Index (SI), along with time efficiency comparisons between manual and automatic contouring.

**Results:**

The auto-segmentation achieved favorable accuracy across multiple structures. For bone marrow, the DSC, HD, JAC, and SI were 0.89 ± 0.05, (2.39 ± 0.90) mm, 0.80 ± 0.11, and 0.87 ± 0.04, respectively. Bladder and femoral heads also showed high concordance, with DSCs exceeding 0.91 and HDs below 2 mm. Auto-segmentation significantly reduced contouring time across all structures; for CTV, the average time decreased from (4151.54 ± 300.23) seconds to(45.82 ± 2.00)seconds (t=-102.10,p< 0.001).From a dosimetric perspective, auto-segmentation achieved comparable CTV coverage to manual methods (P > 0.05), but showed statistically significant improvements in organ-at-risk sparing for bone marrow, small bowel, and rectum (P< 0.05). No clinically relevant differences were detected for bladder or femoral head doses.

**Conclusion:**

The RT-Mind software based on a U-Net architecture demonstrates high accuracy and efficiency in segmenting CTV and OARs in postoperative radiotherapy for cervical cancer, particularly in delineating pelvic bone marrow. It effectively reduces contouring time and inter-observer variability, offering promising clinical applicability.

## Introduction

Cervical cancer is one of the most prevalent malignancies among women worldwide. Due to its unique anatomical location, radiotherapy serves as a critical therapeutic modality, particularly for patients with locally advanced disease, postoperative recurrence, or inoperable tumors (1). In recent years, advancements in radiotherapy techniques, including Intensity-Modulated Radiation Therapy (IMRT) and Volumetric Modulated Arc Therapy (VMAT), have been widely integrated into clinical practice. These technologies enable precise modulation of radiation dose distribution, effectively sparing adjacent normal tissues and thereby significantly reducing radiation-induced toxicity (2, 3).

However, achieving precision therapy fundamentally depends on the accurate definition and delineation of both the Clinical Target Volume (CTV) and Organs-at-Risk (OARs) (4–6). During pelvic radiotherapy for cervical cancer, the pelvic bone marrow (PBM), an essential hematopoietic organ, is particularly vulnerable to radiation-induced damage (7). Such damage to the PBM frequently results in varying degrees of myelosuppression, impairing hematopoietic function, compromising patient immunity, and consequently elevating the risk of adverse events such as infection, anemia, and thrombocytopenia (8, 9). Thus, the rapid and accurate segmentation of PBM has emerged as a key technical challenge in the radiotherapy planning process for cervical cancer patients.

Traditional delineation of pelvic bone marrow (PBM) primarily relies on manual contouring by radiation oncologists based on computed tomography (CT) images. Given the absence of distinct anatomical boundaries, PBM morphology and density can exhibit significant inter-individual variability (10). Clinically, PBM contours are commonly approximated indirectly by delineating regions of bone density within pelvic skeletal structures, including the ilium, sacrum, ischium, pubis, and proximal femur. However, this manual approach is time-consuming, and significant variability in contouring outcomes arises from differences in physician experience, clinical practice patterns, and image contrast quality, potentially compromising treatment precision and consistency. Furthermore, radiation oncologists must carefully balance radiation doses to PBM and adjacent organs-at-risk (OARs) during the contouring process, adding further complexity and workload to treatment planning.

RT-Mind is an auto-segmentation software developed based on convolutional neural networks (CNNs). The underlying model was originally proposed and validated. in the field of cervical cancer radiotherapy. Unlike conventional U-Net architectures and their derivatives (e.g., U-Net++, Attention U-Net, and nnU-Net), the DpnUNet framework incorporated in RT-Mind introduces substantial architectural innovations. Specifically, the encoder of the traditional U-Net is replaced with a Dual Path Network (DPN), which integrates Residual Blocks for feature reuse and Dense Blocks for new feature exploration. This dual-path design enables the network to preserve previously learned spatial representations while efficiently extracting higher-level semantic features, thereby enhancing its ability to delineate structures with complex and low-contrast anatomical boundaries. In addition, RT-Mind incorporates the optimized RTD-Net architecture, an improved variant of the U-Net family, which employs an encoder–decoder structure for multi-scale feature extraction and utilizes cross-layer connections to enhance feature fusion. These architectural improvements collectively strengthen the model’s robustness and accuracy in contouring pelvic structures with intricate anatomical relationships (11).

Given these advantages, the present study aims to evaluate the clinical feasibility of RT-Mind by comparing its auto-segmented contours with those generated manually by radiation oncologists. The comparison focuses on contour overlap, boundary accuracy, and contouring efficiency. Furthermore, the study investigates the potential of RT-Mind to optimize radiotherapy planning, improve dose sparing of pelvic bone marrow (PBM), and ultimately contribute to better clinical outcomes.

## Methods

### Collection and processing of expression data

A total of 55 cervical cancer patients who underwent postoperative radiotherapy from March 2024 to January 2025 were retrospectively enrolled from the Departments of Radiation Oncology at Siyang Hospital and the Affiliated Hospital of Jiangsu University. Patients ranged in age from 27 to 68 years, with a mean age of (46.0 ± 9.37) years. The postoperative pathological subtypes were predominantly squamous cell carcinoma (n=51), with a minority presenting adenocarcinoma (n=4). None of the patients had previously received pelvic radiotherapy, and all had no history of bone marrow disorders.

### Inclusion and exclusion criteria

The inclusion criteria were as follows: (1) histopathologically confirmed cervical cancer with completion of radical surgery; (2) indication for adjuvant pelvic intensity-modulated radiotherapy (IMRT) based on postoperative clinical assessment; (3) no history of hematopoietic dysfunction or underlying diseases known to impair bone marrow function. Patients were excluded if they met any of the following conditions: (1) concurrent pelvic malignancies or a prior history of other cancers; (2) significant pelvic deformities, severe osteoporosis, or advanced degenerative joint diseases; (3) poor-quality postoperative CT images unsuitable for accurate delineation and evaluation; or (4) incomplete clinical data or lack of follow-up information.

### Equipment and imaging protocols

Plain and contrast-enhanced computed tomography (CT) simulation scans were acquired using a Philips Brilliance CT Big Bore 4D scanner (Philips Healthcare, The Netherlands). The obtained CT images were imported into the RT-Mind-WS0.4 automatic segmentation software (Yizhun Medical Technology Co., Ltd., Beijing, China) for delineation of the clinical target volume (CTV) and organs at risk (OARs). Following segmentation, image datasets were exported to the Monaco treatment planning system (Elekta AB, Stockholm, Sweden) for subsequent dosimetric analyses. The RT-Mind auto-segmentation model is built upon an enhanced U-Net (RTD-Net) architecture and was trained using multi-center clinical radiotherapy datasets provided by Beijing YizhiYing Technology Co., Ltd. The training cohort comprised pelvic radiotherapy CT images collected from several tertiary hospitals in China, including both preoperative and postoperative cervical cancer cases. Data augmentation strategies—such as translation, rotation, and scaling—were applied to improve the model’s robustness and adaptability across varying anatomical structures and imaging conditions.

### Manual delineation of regions of interest

Manual delineation of clinical target volumes (CTV) and organs-at-risk (OARs) was performed on the Monaco treatment planning system (Elekta AB, Stockholm, Sweden) by two experienced radiation oncologists according to the guidelines outlined in International Commission on Radiation Units and Measurements (ICRU) Report No. 83. The CTV was defined as areas potentially harboring residual microscopic tumor or at risk for subclinical tumor spread postoperatively, encompassing the primary surgical site, cervical stump, parametrial tissues, vaginal cuff extending inferiorly approximately 3–4 cm based on postoperative pathological findings and patient anatomy, and pelvic lymphatic drainage areas (including common iliac, external iliac, internal iliac, and obturator lymph node regions). Multimodal imaging, including preoperative magnetic resonance imaging (MRI) and positron emission tomography-computed tomography (PET-CT), as well as postoperative histopathological findings, were integrated into the delineation process to enhance target accuracy.

The delineated OARs included the bladder (entire bladder wall, accounting for variations in bladder filling), rectum (complete rectal wall extending from the rectosigmoid junction to the anal verge), bilateral femoral heads (from the cranial tip to the level of the lesser trochanter), pelvic bone marrow (low-density marrow regions extending cranially approximately 2 cm beyond the superior margin of the planning target volume (PTV), including vertebrae L4–L5 and the entire sacrum, inferiorly limited to the level of the ischial tuberosities), and small bowel (all bowel loops within the pelvic cavity, carefully considering their mobility and volumetric changes).

### Automatic delineation of regions of interest

For automatic segmentation, CT simulation images from 55 postoperative cervical cancer patients undergoing intensity-modulated radiotherapy (IMRT) were imported into the RT-Mind software. This software employed a dedicated deep learning model specifically trained for postoperative cervical cancer radiotherapy to automatically segment both the Clinical Target Volume (CTV) and the aforementioned Organs-at-Risk (OARs).All CTV and OAR contours—whether generated manually or automatically—were independently delineated by two senior radiation oncologists. The resulting contours were subsequently reviewed and, if necessary, revised by a chief radiation oncologist to serve as the reference standard for further evaluation. These expert-reviewed contours formed the basis for analyzing the accuracy and clinical applicability of the RT-Mind software. This process was designed to assess the feasibility and precision of using the RT-Mind platform for automatic segmentation in pelvic radiotherapy planning for postoperative cervical cancer patients.

### Evaluation metrics

The manual contours delineated by radiation oncologists were used as the reference standard (ground truth) for evaluating segmentation accuracy. Four quantitative metrics were applied to assess the performance of the U-Net-based automatic segmentation model in delineating the Clinical Target Volume (CTV) and Organs-at-Risk (OARs): Dice Similarity Coefficient (DSC), Hausdorff Distance (HD), Jaccard Index (JAC), and Sensitivity Index (SI).To minimize evaluation bias, a blinded assessment was implemented during the consistency analysis of manual and automatic contours. The reviewing radiation oncologists were unaware of the contour source (manual or automatic), as all contour sets were anonymized and presented in a randomized order.

### Dice similarity coefficient

The Dice Similarity Coefficient (DSC) quantifies the volumetric overlap between automatically and manually delineated regions of interest. Its value ranges from 0 to 1, with higher values indicating greater spatial agreement. A DSC closer to 1 reflects a higher degree of consistency between the automatic and manual contours. $\text{DSC}=\frac{2\times \left({\text{V}}_{\text{ref}}\cap {\text{V}}_{\text{auto}}\right)}{{\text{V}}_{\text{ref}}{\text{V}}_{\text{auto}}}$. In the formula,V_ref_ represents the volume of the region of interest manually delineated by radiation oncologists, while V_auto_ denotes the volume automatically segmented by the software.

### Hausdorff distance

The Hausdorff Distance (HD) measures the maximum distance between the contour point sets of the automatic and manual segmentations in three-dimensional space. It is expressed in millimeters (mm), with smaller values indicating better boundary agreement between the automatic and manual contours. HD(X,Y)=max(h(X,Y),h(Y,X)). In the formula, X denotes the set of points on the automatically segmented contour, and Y represents the set of points on the manually delineated contour $\text{h}\left(\text{X},\text{Y}\right)=\frac{\text{maxmin}}{\text{x}\in \text{Xy}\in \text{Y}}∥\text{x}-\text{y}∥$.

### Jaccard index

The Jaccard Index (JAC) measures the ratio of the intersection to the union of the automatically and manually delineated contours. Its value ranges from 0 to 1, with values closer to 1 indicating a higher degree of similarity between the two contour sets $\text{JAC}=\frac{{\text{V}}_{\text{ref}}\cap {\text{V}}_{\text{auto}}}{{\text{V}}_{\text{ref}}\cup {\text{V}}_{\text{auto}}}$.

### Sensitivity index

The Sensitivity Index (SI) represents the proportion of the intersection between the automatically and manually delineated regions relative to the manually delineated region. Its value ranges from 0 to 1, with higher values indicating a greater extent to which the automatic segmentation covers the manually defined region.

### Statistical analysis

Statistical analysis was performed using SPSS version 27.0 (IBM Corp., Armonk, NY, USA). Continuous variables were expressed as mean ± standard deviation. Paired t-tests were used to compare the time efficiency and dosimetric parameters between the manual and automatic segmentation methods. A p-value< 0.05 was considered statistically significant.

## Results

### Comparison of segmentation efficiency between automatic and manual contouring of CTV and OARs in postoperative cervical cancer patients

**Figure 1:**
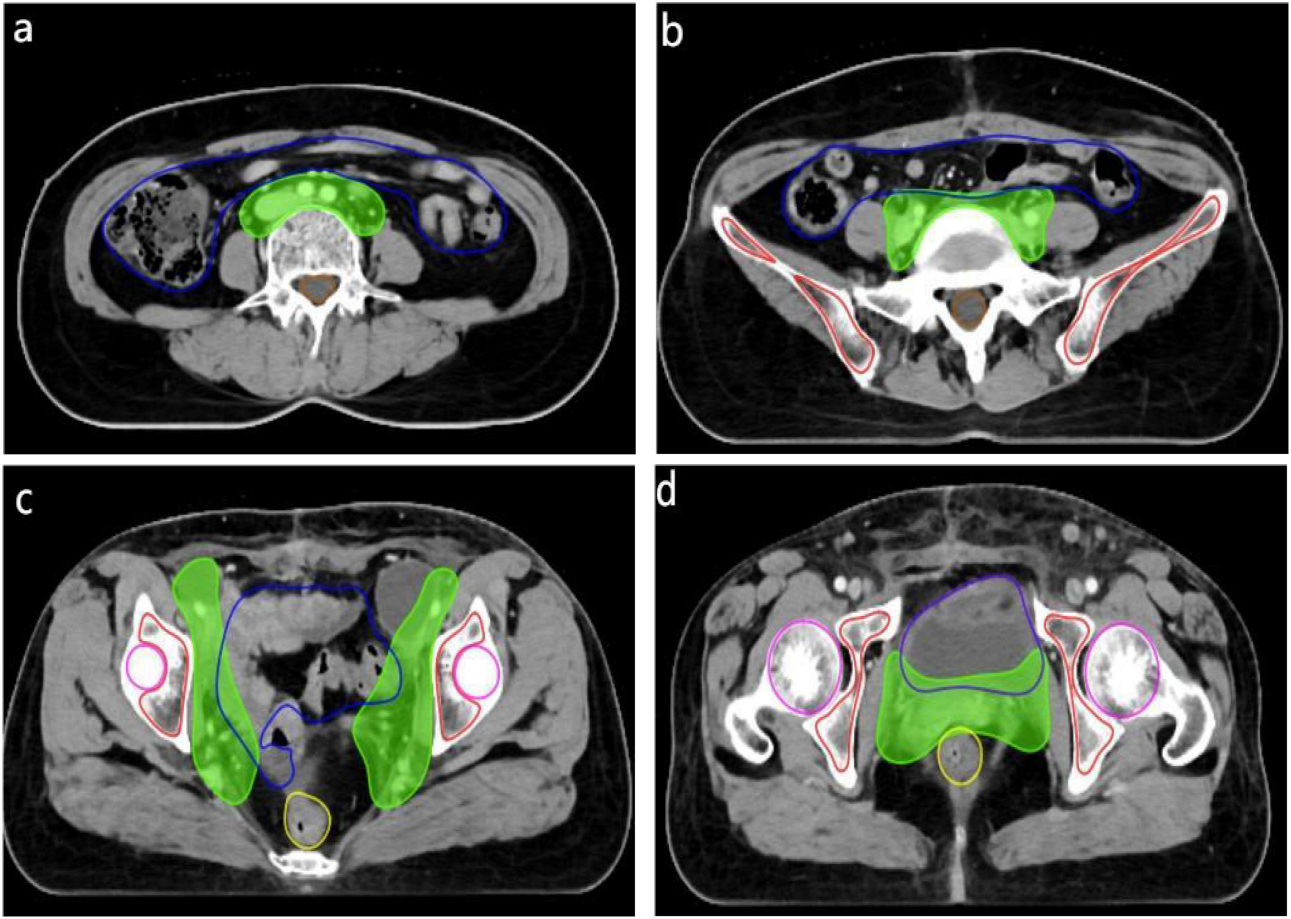
Representative axial CT images of the abdomen and pelvis at different anatomical levels: **(a)** axial CT image at the level immediately inferior to the kidneys (infrarenal level); **(b)** axial CT image at the pelvic inlet (iliac level); **(c)** axial CT image at the level of the true pelvis (acetabular level); **(d)** axial CT image at the lower pelvis, at the level of the urinary bladder and femoral heads.

**Figure 2:**
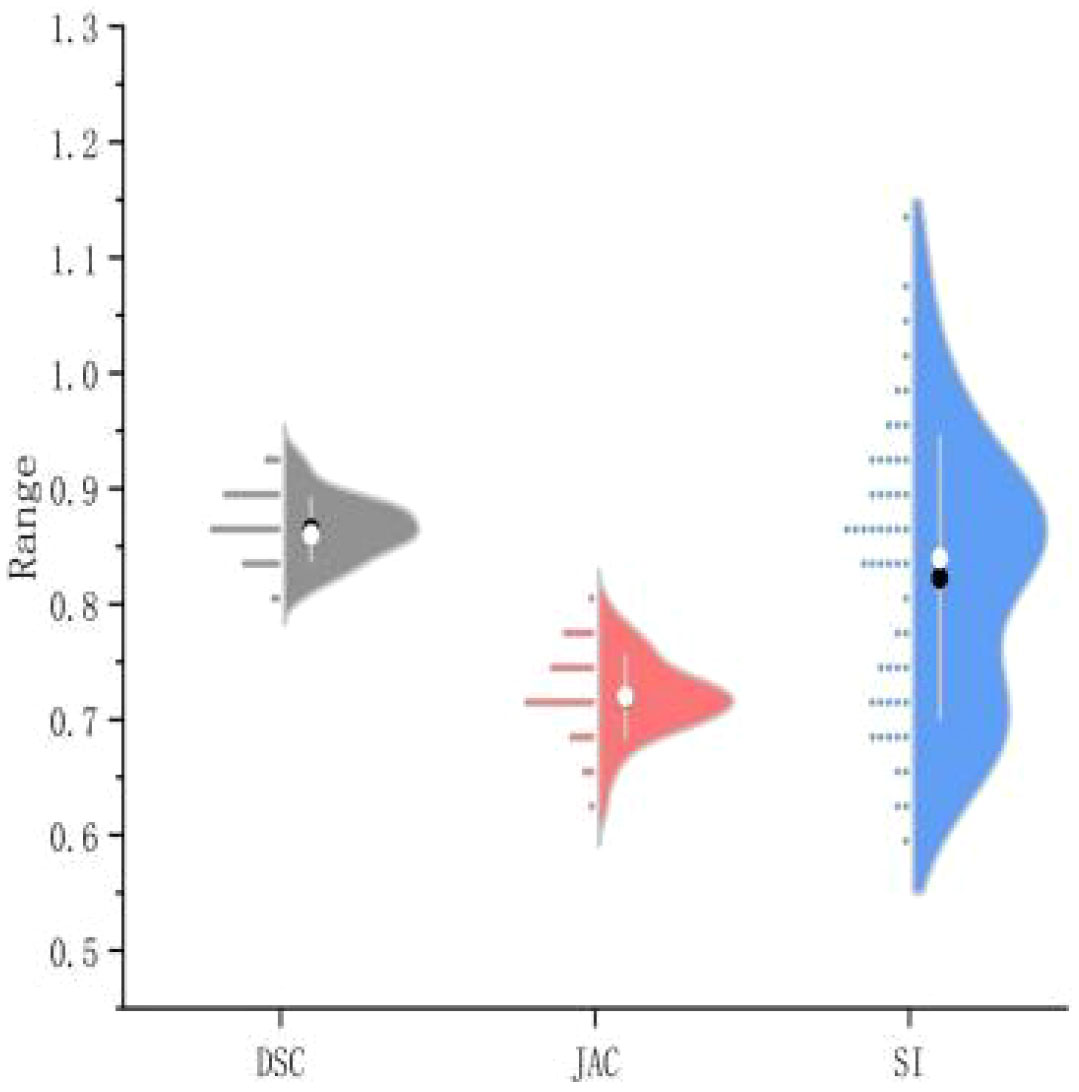
Half-violin plot of CTV segmentation efficiency.

**Figure 3:**
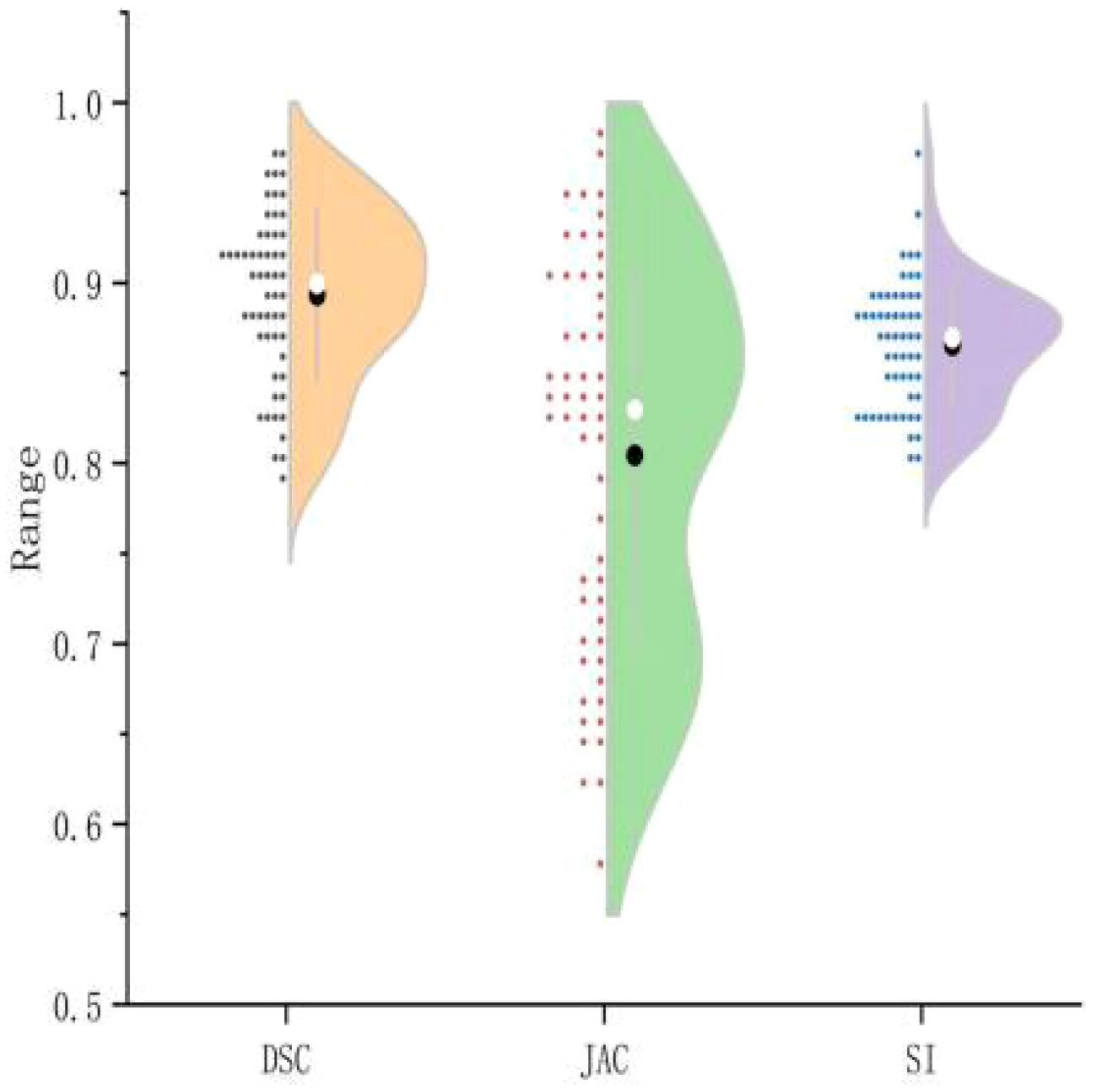
Half-violin plot of bone marrow segmentation efficiency.

This study assessed the performance of a U-Net-based automated segmentation software for delineating Clinical Target Volume (CTV) and Organs-at-Risk (OARs) in postoperative radiotherapy for cervical cancer, with detailed results presented in **Table 1**. Representative transverse CT images illustrating the automated segmentation are provided in **Figure 1**, and half-violin plots for DSC, JAC, and SI of CTV and bone marrow segmentation are depicted in **Figures 2** and **3**. The Dice Similarity Coefficient (DSC) for CTV was (0.86 ± 0.03), Hausdorff Distance (HD) was (9.17 ± 3.32) mm, Jaccard coefficient (JAC) was (0.72 ± 0.04), and Sensitivity Index (SI) was (0.82 ± 0.12). Among the OARs, bladder segmentation demonstrated the highest accuracy with a DSC of (0.94 ± 0.02) and JAC of (0.87 ± 0.07), indicating excellent agreement with manual contours. Automated segmentation accuracy was also high for the left and right femoral heads, with DSC values of (0.92 ± 0.03) and (0.91 ± 0.04), respectively, and HD less than 2 mm. Rectum and bowel loops had DSC values of 0.82, JAC values of (0.65 ± 0.10) and (0.70 ± 0.13), and HD values of (4.94 ± 1.30) mm and (12.66 ± 2.26) mm, respectively; although slightly lower than other structures, their segmentation accuracy remained clinically acceptable. Bone marrow segmentation exhibited good performance with a DSC of (0.89 ± 0.05) and HD of (2.39 ± 0.90) mm.

**Table 1:** Accuracy evaluation of auto-segmentation versus manual delineation for CTV and OARs in postoperative cervical cancer patients ( $\bar{x}$ ± *s*).

Parameters	DSC	HD (mm)	JAC	SI
CTV	0.86 ± 0.03	9.17 ± 3.32	0.72 ± 0.04	0.82 ± 0.12
Rectum	0.82 ± 0.05	4.94 ± 1.30	0.65 ± 0.10	0.82 ± 0.06
Bladder	0.94 ± 0.02	7.52 ± 1.09	0.87 ± 0.07	0.89 ± 0.06
Bowel bag	0.82 ± 0.06	12.66 ± 2.26	0.70 ± 0.13	0.77 ± 0.09
Bone marrow	0.89 ± 0.05	2.39 ± 0.90	0.80 ± 0.11	0.87 ± 0.04
Left femoral head	0.92 ± 0.03	1.81 ± 0.70	0.83 ± 0.06	0.86 ± 0.04
Right femoral head	0.91 ± 0.04	1.77 ± 0.73	0.82 ± 0.09	0.85 ± 0.09

### Comparison of contouring efficiency between automatic and manual segmentation of CTV and OARs in postoperative cervical cancer patients

Regarding segmentation time, as shown in **Table 2**, automated segmentation significantly reduced delineation time compared to manual contouring across all structures. The manual contouring time for Clinical Target Volume (CTV) was (4151.54 ± 300.23) seconds, whereas automated segmentation required only (45.82 ± 2.00) seconds, representing a statistically significant difference (t = -102.10, p< 0.001). For organs-at-risk (OARs), automated delineation of the bladder, rectum, and bowel loops was consistently completed within 50 seconds, markedly less than manual contouring times, with all differences statistically significant (p< 0.001).

**Table 2:** Comparison of efficiency between manual delineation and auto-segmentation for regions of interest (ROIs) ( $\bar{x}$ ± s).

Parameters	Manual contouring(s)	Automatic delineation(s)	*t*	*p*
CTV	4151.54 ± 300.23	45.82 ± 2.00	-102.10	<0.001
Rectum	198.15 ± 20.73	45.46 ± 2.17	-61.00	<0.001
Bladder	223.67 ± 13.70	44.42 ± 2.57	-119.37	<0.001
Bowel bag	1819.01 ± 180.86	47.97 ± 2.62	-73.69	<0.001
Bone marrow	2119.97 ± 233.00	49.69 ± 2.51	-66.61	<0.001
Left femoral head	207.55 ± 20.36	43.28 ± 2.39	-67.80	<0.001
Right femoral head	202.56 ± 19.92	43.63 ± 2.01	-65.82	<0.001

### Comparison of dosimetric parameters of CTV and OARs between automatic and manual segmentation in postoperative cervical cancer patients

**Figure 4:**
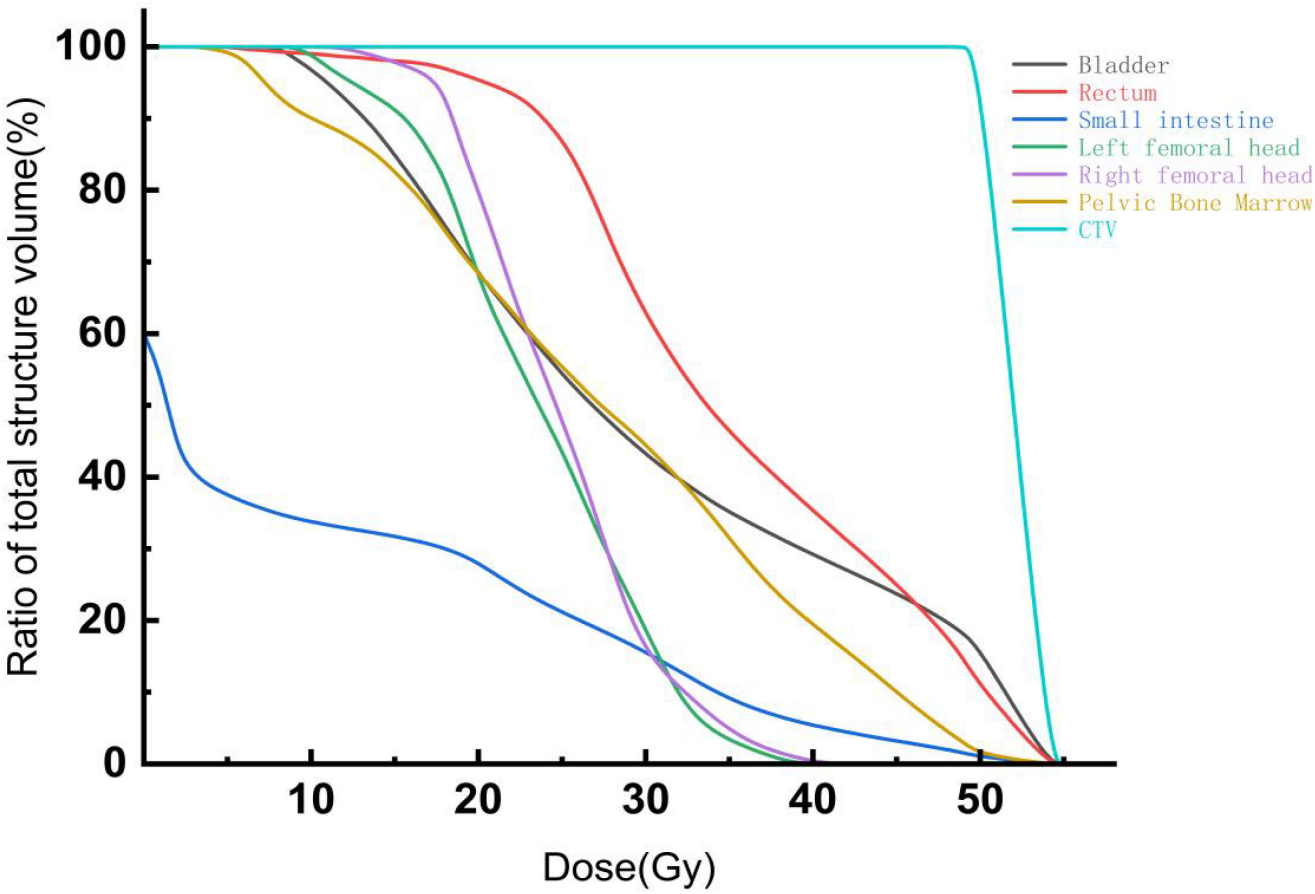
DVH curves of CTV and organs-at-risk (OARs).

Dose-volume histograms for Clinical Target Volume (CTV) and organs-at-risk (OARs) for the two segmentation methods are presented in **Figure 4**. As shown in **Table 3**, there were no statistically significant differences (P > 0.05) between automatic and manual segmentation groups regarding dosimetric parameters for CTV, including Dmax, Dmin, Dmean, conformity index (CI), and homogeneity index (HI), indicating good agreement in target dose distributions. **Table 4** demonstrates that the automatic segmentation group had significantly lower values in bone marrow dosimetric parameters, such as V20%, V30%, V40%, and Dmean, compared to manual segmentation (P< 0.05), suggesting superior performance of automatic segmentation in bone marrow protection. Furthermore, statistically significant differences were also observed in dosimetric parameters for small bowel V40% and Dmax, and rectum V40% and Dmean (P< 0.05). Conversely, parameters for bladder and femoral heads showed no significant differences (P > 0.05), further supporting the feasibility of automatic segmentation in effectively managing OARs dose constraints.

**Table 3:** Dosimetric comparison of treatment target in the two groups ( $\bar{x}$ ± *s*).

Parameters	Manual contouring	Automatic delineation	t	p
Dmax (Gy)	55.68 ± 0.34	55.51 ± 0.45	2.07	0.062
Dmin (Gy)	51.49 ± 1.15	51.56 ± 0.11	-0.41	0.683
Dmean (Gy)	48.61 ± 0.31	48.59 ± 0.28	0.45	0.650
CI	0.872 ± 0.024	0.875 ± 0.020	-0.73	0.466
HI	0.072 ± 0.012	0.071 ± 0.008	0.61	0.545

**Table 4:** Dosimetric comparison of OARs in the two groups.

OARs	Variable	Manual contouring	Automatic delineation	*t*	*p*
Bladder	V20 (%)	68.30 ± 1.04	68.19 ± 0.50	0.90	0.370
	V30 (%)	43.36 ± 4.92	43.77 ± 1.60	-0.58	0.563
	V40 (%)	29.24 ± 2.76	28.97 ± 2.29	-0.97	0.337
	Dmax (Gy)	54.17 ± 0.15	54.38 ± 0.38	-1.57	0.123
	Dmean (Gy)	29.97 ± 0.89	30.03 ± 0.40	-0.51	0.612
Rectum	V20 (%)	95.56 ± 2.13	95.77 ± 2.67	-0.41	0.683
	V30 (%)	62.38 ± 4.43	62.03 ± 1.34	0.55	0.584
	V40 (%)	36.36 ± 4.56	40.50 ± 2.51	-6.90	<0.001
	Dmax (Gy)	54.94 ± 1.96	54.82 ± 0.34	0.44	0.661
	Dmean (Gy)	35.86 ± 1.87	35.16 ± 0.61	2.19	0.033
Small intestine	V20 (%)	29.03 ± 6.93	28.02 ± 13.23	0.57	0.572
	V30 (%)	15.98 ± 7.81	16.23 ± 10.93	-0.16	0.872
	V40 (%)	4.25 ± 6.73	7.18 ± 7.73	-2.24	0.030
	Dmax (Gy)	51.49 ± 0.71	53.90 ± 0.37	-21.84	<0.001
	Dmean (Gy)	10.78 ± 0.14	10.72 ± 0.34	1.16	0.253
Left femoral head	V20 (%)	66.92 ± 8.89	67.83 ± 10.67	-0.50	0.618
	V30 (%)	18.57 ± 8.11	19.59 ± 8.16	-0.66	0.511
	Dmax (Gy)	40.47 ± 3.54	39.84 ± 2.70	1.06	0.294
	Dmean (Gy)	23.66 ± 3.91	23.16 ± 1.35	0.92	0.359
Right femoral head	V20 (%)	79.98 ± 9.26	80.87 ± 9.90	-0.49	0.629
	V30 (%)	16.43 ± 7.59	15.33 ± 6.45	0.75	0.455
	Dmax (Gy)	42.62 ± 2.57	42.56 ± 2.47	0.12	0.901
	Dmean (Gy)	25.11 ± 4.85	24.88 ± 1.56	0.32	0.749
Bone marrow	V20 (%)	68.03 ± 2.75	71.84 ± 4.74	-5.04	<0.001
	V30 (%)	44.08 ± 2.15	46.55 ± 3.55	-4.00	<0.001
	V40 (%)	18.55 ± 5.11	20.66 ± 3.68	-2.47	0.017
	Dmax (Gy)	53.17 ± 1.15	53.72 ± 1.61	-1.89	0.064
	Dmean (Gy)	27.83 ± 2.52	29.76 ± 2.97	-3.62	<0.001

## Discussion

With advancements in radiotherapy precision and rapid developments in adaptive radiation therapy (ART), research on automated segmentation of clinical target volumes (CTV) and organs-at-risk (OARs) has become increasingly crucial (12–14). Deep learning-based automated segmentation methods currently surpass traditional atlas-based matching approaches in terms of accuracy and efficiency, significantly reducing segmentation time from tens of minutes to mere seconds. This efficiency reduces preparation time, minimizes tumor progression risks associated with delays, and facilitates broader clinical implementation of adaptive radiation therapy (15). However, the feasibility and stability of deep learning-based segmentation software in various postoperative cervical cancer clinical scenarios still require further validation and optimization.

In this study, RT-Mind, an automatic segmentation software developed using a convolutional neural network (CNN), was evaluated for automatic delineation of CTV and OAR contours in postoperative cervical cancer radiotherapy. Segmentation accuracy and clinical feasibility were assessed through comparisons between automated results and manual delineations by experienced radiation oncologists. The RT-Mind software demonstrated excellent segmentation performance across multiple anatomical structures, particularly achieving high consistency in pelvic bone marrow delineation. According to Zijdenbos et al. (16), a Dice Similarity Coefficient (DSC) greater than 0.7 indicates favorable segmentation accuracy. In the current study, the DSC for pelvic bone marrow reached 0.89, with a Hausdorff Distance (HD) of 2.3 mm, suggesting a high accuracy in automatic identification of the bone marrow region by RT-Mind. This high accuracy may be attributed to the implementation of skip-connection mechanisms between encoder and decoder layers in the CNN architecture, enabling comprehensive integration of multi-scale image features and enhancing recognition capability in anatomically ambiguous regions.

For OARs with clear boundaries and regular anatomical structures, such as the bladder and femoral heads, the automated segmentation achieved the highest overlap, with DSC values exceeding 0.91. This indicates the model’s strong adaptability in high-contrast imaging areas with distinct anatomical boundaries. In contrast, segmentation results for organs like the rectum and bowel loops, characterized by complex anatomy, variable positioning, and lower CT image contrast, were slightly inferior, yet still clinically acceptable. Furthermore, RT-Mind adopts a 2.5D input strategy in which three consecutive CT slices are combined as input channels. This approach enables the model to capture inter-slice spatial context while maintaining computational efficiency, making it more suitable for pelvic CT imaging in cervical cancer, where relatively thick slice spacing is common. In the benchmark study by Liu et al, the DpnUNet architecture demonstrated superior performance compared with conventional U-Net and CabUNet models, achieving a mean Dice similarity coefficient of 0.86 and a 95th percentile Hausdorff distance of 5.34 mm for CTV segmentation—results that are highly consistent with the findings of the present study (17).

In clinical radiation therapy, pelvic bone marrow (PBM), as a critical hematopoietic organ for cervical cancer patients, is highly sensitive to radiation doses and represents a key organ-at-risk (OAR) requiring careful protection. Exposure of PBM to high radiation doses can lead to myelosuppression, manifesting clinically as leukopenia, anemia, and thrombocytopenia, among other hematologic toxicities (18–21).Zhu et al. investigated the relationship between changes in bone marrow cell counts and the mean pelvic bone marrow (PBM) dose in a male lymphoma mouse model of cervical cancer. Their results demonstrated that for every 1 Gy increase in mean PBM dose, weekly neutrophil and white blood cell counts decreased by 9.6 and 7.8 μL^−1^, respectively, indicating a clear dose-dependent association with acute hematologic toxicity. In a multivariate logistic regression analysis, Albuquerque et al. identified PBM V20 as an independent predictor of ≥ Grade 2 hematologic toxicity (r = 0.8, P< 0.001). Further analysis revealed that patients with PBM V20 > 80% had a significantly increased risk of developing ≥ Grade 2 hematologic toxicity (OR = 4.5, P< 0.05) (22, 23).Such adverse effects compromise patient immunity and overall tolerance, potentially resulting in treatment interruptions, reduced therapeutic efficacy, and impaired long-term prognosis (24). Therefore, precise delineation of PBM is clinically essential for optimizing dose distribution, enhancing treatment safety, and improving outcomes. However, traditional manual contouring methods are time-consuming, subjective, and associated with considerable inter-observer variability (25).

This study demonstrated that RT-Mind significantly reduces contouring time; the average delineation time for the Clinical Target Volume (CTV) decreased from 4151 seconds with manual delineation to less than 50 seconds using automatic segmentation, representing a time-saving of over 98% and highlighting substantial efficiency improvements. Additionally, rapid automated segmentation indirectly reduces delays between patient positioning and treatment initiation. The rapid and precise delineation of PBM using tools such as RT-Mind not only enhances workflow efficiency but also improves treatment timeliness and accuracy.

Despite its notable advantages in efficiency and consistency, the clinical implementation of U-Net-based automatic segmentation faces several challenges. For instance, PBM boundaries may have limited visibility on CT images, particularly in patients with anatomical abnormalities or postoperative structural remodeling, potentially compromising accurate automated identification. Furthermore, automated segmentation outcomes still require manual verification and occasional revision by radiation oncologists, particularly in complex clinical scenarios involving tumor infiltration, postoperative anatomical alterations, or pelvic deformities.

A major limitation of this study is that it was conducted as a single-center, retrospective analysis with a relatively small sample size. The patient population and contouring workflow reflect the clinical practice of our institution, which may affect the generalizability of the findings. Therefore, the applicability of the auto-segmentation model across different centers, radiotherapy workflows, and imaging settings requires further validation. Future work should involve multi-center, prospective studies with larger cohorts to enhance the representativeness and statistical power of the results and to more comprehensively assess the clinical value and scalability of the auto-segmentation approach.

## Conclusion

This study evaluated the efficacy of RT-Mind, an automatic segmentation software based on the U-Net convolutional neural network, for the delineation of Clinical Target Volume (CTV) and organs-at-risk (OARs), including pelvic bone marrow (PBM), in postoperative cervical cancer radiotherapy. Results demonstrated that RT-Mind achieved high accuracy in segmenting structures such as the bladder, femoral heads, and bone marrow, significantly outperforming manual delineation in terms of time efficiency and exhibiting dosimetric advantages. Although some inaccuracies were noted in soft-tissue structures, such as bowel loops, the overall clinical acceptability remained high. In conclusion, RT-Mind facilitates rapid, standardized delineation for postoperative cervical cancer radiotherapy and shows substantial promise for clinical application.

## Data Availability

The raw data supporting the conclusions of this article will be made available by the authors, without undue reservation.
